# Cross-phosphorylation of bacterial serine/threonine and tyrosine protein kinases on key regulatory residues

**DOI:** 10.3389/fmicb.2014.00495

**Published:** 2014-09-17

**Authors:** Lei Shi, Nathalie Pigeonneau, Vaishnavi Ravikumar, Paula Dobrinic, Boris Macek, Damjan Franjevic, Marie-Francoise Noirot-Gros, Ivan Mijakovic

**Affiliations:** ^1^SysBio, Department of Chemical and Biological Engineering, Chalmers University of TechnologyGöteborg, Sweden; ^2^UMR1319 Micalis, Institut National de Recherche AgronomiqueJouy-en-Josas, France; ^3^Proteome Center Tübingen, Interfaculty Institute for Cell Biology, University of TübingenTübingen, Germany; ^4^Division of Biology, Faculty of Science, Zagreb UniversityZagreb, Croatia

**Keywords:** protein phosphorylation, bacterial protein kinase, protein kinase cross-talk, phosphorylation cascade, kinase activation

## Abstract

Bacteria possess protein serine/threonine and tyrosine kinases which resemble eukaryal kinases in their capacity to phosphorylate multiple substrates. We hypothesized that the analogy might extend further, and bacterial kinases may also undergo mutual phosphorylation and activation, which is currently considered as a hallmark of eukaryal kinase networks. In order to test this hypothesis, we explored the capacity of all members of four different classes of serine/threonine and tyrosine kinases present in the firmicute model organism *Bacillus subtilis* to phosphorylate each other *in vitro* and interact with each other *in vivo*. The interactomics data suggested a high degree of connectivity among all types of kinases, while phosphorylation assays revealed equally wide-spread cross-phosphorylation events. Our findings suggest that the Hanks-type kinases PrkC, PrkD, and YabT exhibit the highest capacity to phosphorylate other *B. subtilis* kinases, while the BY-kinase PtkA and the two-component-like kinases RsbW and SpoIIAB show the highest propensity to be phosphorylated by other kinases. Analysis of phosphorylated residues on several selected recipient kinases suggests that most cross-phosphorylation events concern key regulatory residues. Therefore, cross-phosphorylation events are very likely to influence the capacity of recipient kinases to phosphorylate substrates downstream in the signal transduction cascade. We therefore conclude that bacterial serine/threonine and tyrosine kinases probably engage in a network-type behavior previously described only in eukaryal cells.

## Introduction

In eukarya, protein kinases are known to participate in regulatory networks involved in cell cycle control, signal transduction, and other complex regulatory phenomena (Colinge et al., 2014). Most of the characterized eukaryal kinases exhibit two key functional features: each protein kinase phosphorylates a number of different protein substrates, and kinases phosphorylate and activate each other, thus participating in elaborate phosphorylation cascades (Marshall, 1994). Bacteria, whose cellular makeup is considered to be simplified and optimized for rapid bursts of growth, were usually thought not to possess such complicated kinase networks. The main sensing and signal transduction devices in bacteria are the so-called two-component systems, based on histidine/aspartate kinases (Goulian, 2010). The two component systems are very rapid signal transmission devices, linking environmental stimuli to gene expression. However, they operate mostly as linear signaling pathways, with essentially no cross-talk among different two-component systems (Podgornaia and Laub, 2013). Even in extended bacterial signaling systems, involving sequential phosphorylation of several two-component-like kinases, the flow of information remains linear (Burbulys et al., 1991). In addition to two-component systems, bacteria also possess serine/threonine (Pereira et al., 2011) and tyrosine protein kinases (Shi et al., 2014). While most bacterial serine/threonine kinases share the origin with their orthologs in eukarya, the bacterial tyrosine kinases do not (Shi et al., 2014). Nevertheless, they all exhibit some properties similar to their eukaryal counterparts. The capacity of bacterial serine/threonine and tyrosine kinases to phosphorylate multiple substrates has been clearly established. Examples of well-characterized bacterial kinases with multiple substrates include a number of Hanks-type serine/threonine kinases from *Mycobacterium tuberculosis* (Grundner et al., 2005; Molle and Kremer, 2010; Baer et al., 2014) and *Bacillus subtilis* (Absalon et al., 2009; Pietack et al., 2010; Ravikumar et al., 2014), as well as the tyrosine kinase PtkA from *B. subtilis* (Petranovic et al., 2009; Jers et al., 2010; Derouiche et al., 2013). By contrast, the capacity of bacterial protein kinases to phosphorylate each other is far less documented. Cross-phosphorylation among some Hanks-type serine/threonine kinases has recently been reported in *M. tuberculosis* (Baer et al., 2014). There is also evidence that Hanks-type serine/threonine kinases from *B. subtilis* can phosphorylate and activate a two-component histidine/aspartate kinase DegS (Jers et al., 2011). We have previously hypothesized that bacterial serine/threonine and tyrosine kinases, given their functional similarity to eukaryal kinases, might also constitute signal integration hubs by phosphorylating each other (Cousin et al., 2013). In order to test this hypothesis, we have elected to use *B. subtilis* as the system of study, due to the fact that this model organism possesses four distinct well characterized classes of bacterial serine/threonine and tyrosine kinases. These include two tyrosine kinases: PtkA (Jers et al., 2010) and PtkB (EpsB) (Gerwig et al., 2014); three Hanks-type serine/threonine kinases: PrkC (Madec et al., 2002), PrkD (Kobir et al., 2014), and YabT (Bidnenko et al., 2013); the twin-function kinase/phosphorylase HprK/P involved in carbon catabolite regulation (Hanson et al., 2002); and the three two-component-like serine/threonine kinases: RsbT (Kang et al., 1998), RsbW (Yang et al., 1996), and SpoIIAB (Min et al., 1993). While all of these kinases have been characterized to varying degrees with respect to their physiological role and substrate phosphorylation, their capacity to phosphorylate each other has not been tested previously. Using *in vitro* phosphorylation assays with purified proteins, we demonstrated an extensive network of cross-phosphorylation events involving all four classes of kinases. This cross-talk was also supported by interactomics data. In select cases, we have determined the residues phosphorylated on the “recipient” kinases by various “donor” kinases. The identity of these residues clearly suggests functional importance of cross-phosphorylation events, influencing the activity of the “recipient” kinases.

## Materials and methods

### Protein synthesis and purification

The following kinase and substrate genes were PCR-amplified using the *B. subtilis* 168 genomic DNA as template and the primers listed in Table 1: *rsbT, hprK, rsbW, ptkA, prkD, yabT, prkC, spoIIAB, rsbV, spoIIAA*, and *rsbS*. Site-directed mutagenic PCR (mutagenic primers listed in Table 1) was performed as described previously (Mijakovic et al., 2003), in order to inactivate the catalytic sites of kinases by replacing the catalytic residues: *rsbT* N49A, *hprK* K159M, *rsbW* N53A, *ptkA* K59D, *prkD* K54D, *yabT* K55D, *prkC* K40D, and *spoIIAB* N50A. PCR products were inserted in the plasmid pQE-30 (Qiagen) to produce the 6xHis-tagged fusions of proteins. Strep-tagged versions of proteins were obtained using a pQE-30 vector with His6-tag replaced by a strep-tag (Jers et al., 2010). *Escherichia coli* K12 NM522 and M15 (expressing chaperonins GroEL/GroES) were used for vector construction and protein synthesis, respectively. Cells were routinely grown in LB medium supplemented with appropriate antibiotics when necessary (ampicillin 100 μg/ml and kanamycin 25 μg/ml). Protein synthesis and purification were carried out as described previously (Mijakovic et al., 2003). Briefly, induction was performed at OD_600_ = 0.6 by adding 1 mM IPTG. Cells were harvested 3 h later and disrupted by sonication. The 6xHis- or Strep-tagged proteins were purified from crude extracts using Ni-NTA (Qiagen), or Strep Tactin affinity chromatography (Novagen), respectively. For the insoluble proteins, PrkD K54D and YabT K55D, the inclusion bodies were dissolved in the buffer containing 6 M guanidine hydrochloride, 50 mM Tris pH 7.5, 100 mM NaCl, 5 mM MgCl_2_, and 5% glycerol. The purification was performed as mentioned before but in the buffer with additional 6 M guanidine hydrochloride. To refold the proteins, the concentration of guanidine hydrocloride was lowered to 0.2 M. Purified proteins were desalted on PD-10 columns (GE Healthcare), and stored at −80°C in 10% glycerol.

**Table 1:** **List of PCR primers used in this study**.

**Name**	**Sequence**
*rsbT fwd*	cgcggatccatgaacgaccaatcctgtgtaag
*rsbT rev*	aaaactgcagctaccgaagccatttgatggcttg
*hprk fwd*	cgcggatccatggcaaaggttcgcacaaaag
*hprk rev*	aaaactgcagctattcttcttgttcaccgtcttc
*rsbW fwd*	cgcggatccatgaagaataatgctgattac
*rsbW rev*	aaaactgcagttagttagtttcgtagtttttga
*ptkA fwd*	cgcggatccatggcgcttagaaaaaacaga
*ptkA rev*	aaaactgcagttatttttgcatgaaattgtcc
*prkD fwd*	cgggatccatggcattaaaacttctaaaaaaactgc
*prkD rev*	aaaactgcagttatgtgaccgattgaatggcccg
*yabT fwd*	cgcagatctatgatgaacgacgctttgacgagt
*yabT rev*	ggactgcagtcacccacccgacttagccggtttct
*prkC fwd*	gaagatctatgctaatcggcaagcggatcagcgggcg
*prkC rev*	aaaactgcagttacaaaacccacggccacttttttctttttgccg
*spoIIAB fwd*	cgcggatccatgaaaaatgaaatgcaccttg
*spoIIAB rev*	aaaactgcagttaattacaaagcgctttgct
*rsbV fwd*	cgcggatccatgaatataaatgttgatgtg
*rsbV rev*	aaaactgcagtcattgcactccaccttct
*spoAA fwd*	cgcggatccatgagccttggaattgacatg
*spoAA rev*	aaaactgcagtcatgatgccaccccca
*rsbS fwd*	cgcggatccatgagacatccgaaaatcccga
*rsbS rev*	aaaactgcagctattcccccaattcccgctt
*rsbT N49A fwd*	ttagccagggctatttatttatatgccggcaaagggcagattg
*rsbT N49A rev*	taaataaatagccctggctaattctgaaatagccgttgtaattc
*hprk K159M fwd*	cggcgtcggaatgagcgaaacagcgctagagcttgtgaaaagag
*hprk K159M rev*	ctgtttcgctcattccgacgccgctttttcctgtgatcagcacg
*rsbW N53A fwd*	gcgtgcacagctgcggttcagcacgcttacaaagaagataaa
*rsbW N53A rev*	gctgaaccgcagctgtgcacgcctcactgactgcgattttc
*ptkA K59D fwd*	ggggaaggagattcaacaacggccgccaacctggctgtc
*ptkA K59D rev*	cgttgttgaatctccttcccccggacaagccgatgtaat
*prkD K54D fwd*	ttatgtcttagatcagcttcggccgacaaaagccaaaaag
*prkD K54D rev*	gccgaagctgatctaagacataaggtgtttgagctagg
*YabT K55D fwd*	tgttgccttagatgtgagtgatgacagcctgtctattac
*YabT K55D rev*	catcactcacatctaaggcaacatgtccatctgatgtt
*prkC K40D fwd*	agtcgcaattgatatcctgcggtttgactatgcaaatg
*prkC K40D rev*	caaaccgcaggatatcaattgcgacttcacggtctagaatg
*spoIIAB N50A fwd*	gctgtcacggctgcgattatccatggatatgaagagaactgtg
*spoIIAB N50A rev*	gataatcgcagccgtgacagcctctgacacgactgttttgat

### *In vitro* kinase cross-phosphorylation phosphorylation assay

Phosphorylation reactions were incubated in a buffer containing: 50 mM Tris pH 7.5, 100 mM NaCl, 5 mM MgCl_2_, 5% glycerol, 50 μM ATP, and 20 μCi/mmol [γ-32P]-ATP. Each phosphorylation reaction contained one wild type active kinase and one catalytically deficient kinase. The assay was assembled using the following final protein concentrations: 3 μM RsbT WT, 3 μM HprK WT, 5 μM RsbW WT, 5 μM PtkA WT with 5 μM TkmA, 2 μM PrkD WT, 1 μM YabT WT, 1 μM PrkC WT, 5 μM SpoIIAB WT, 9 μM RsbT N49A, 4 μM HprK K159M, 9 μM RsbW N53A, 3 μM PtkA K59D, 3 μM PrkD K54D, 2 μM YabT K55D, 3 μM PrkC K40D, 9 μM SpoIIAB N50A. Phosphorylation reactions were incubated for 1 h at 37°C, stopped by boiling at 100°C, and samples were separated on an 8–12% SDS-polyacrylamide gel. Autoradiography signals were revealed using the FUJI phosphoimager.

### *In vitro* phosphorylation of HPr

Proteins (2 μM PrkD, 2 μM HprK/P, 6 μM HPr) were incubated in a buffer containing 50 mM Tris pH 7.5, 100 mM NaCl, 5 mM MgCl2, 5% glycerol, and 1 mM ATP to perform the phosphorylation reactions. The reactions were incubated for 1 h at 37°C, stopped by boiling at 100°C, and samples were separated on an 8–12% SDS-polyacrylamide gel. Signals from phosphorylated protein have been revealed by Pro-Q® Diamond phosphoprotein stain (Life Technologies). After two fixation steps in a solution containing 50% methanol and 10% acetic acid (30 min each), the gel was stained by the Pro-Q® Diamond phosphoprotein stain for 90 min. The gel was de-stained by three 30-min washes in a solution containing 20% acetonitrile and 50 mM sodium acetate, pH 4.0. The gel was washed twice with ultrapure water for 5 min, before scanning.

### Identification of phosphorylated residues by mass spectrometry

*In vitro* phosphorylation reactions of PrkD K54D, YabT K55D, PtkA K59D, SpoIIAB N50A, and HprK K159M phosphorylated by PrkC, PtkA, PrkC, PrkD, and PrkD, respectively, were performed as described above, with the only difference of using only non-radioactive ATP. Denaturation of the samples was performed by buffer exchange to 6 M urea and 2 M thiourea in 10 mM Tris-HCl pH 8.0. Mass spectrometry analysis of phosphorylation sites was performed essentially as described previously (Derouiche et al., 2013). Briefly, in-solution digestion with trypsin was followed by phosphopeptide enrichment. Phosphopeptide analysis was performed on a Proxeon Easy-LC system (Proxeon Biosystems) coupled to an LTQ-Orbitrap-XL (Thermo Fisher Scientific) equipped with a nanoelectrospray ion source (Proxeon Biosystems). The five most intense precursor ions were fragmented by activation of neutral loss ions at −98, −49, and −32.6 relative to the precursor ion (multistage activation). Acquired MS spectra were processes with MaxQuant software package (version 1.2.2.9). False discovery rates at peptide, phosphorylation site, and protein group level were set to 1%. Within the modified peptide, phosphorylation events detected with localization probability of at least 0.75 were considered as assigned to a specific residue.

### Yeast two-hybrid assays

Kinase-kinase binary interactions between various kinases and phosphatases were assessed essentially as described previously (Noirot-Gros et al., 2002). The genes encoding the BY-kinases (PtkA, PtkA) Hanks-type serine/threonine-kinases (PrkC, PrkD, YabT), two-component-like serine-kinases (SpoIIAB, RsbT, and RsbW), BY-kinase modulators (TkmA, TkmB), and the phosphotyrosine—and phosphoserine/threonine protein phosphatases (PtpZ, SpoIIE) were inserted in the pGBDU and pGAD yeast two-hybrid vectors to generate in-frame fusions with the DNA-binding domain (BD) and the activating domain (AD), respectively, of Gal4 (James et al., 1996). The pGBD and pGAD constructs were used to transform yeast (a) and (α) strains, respectively. Binary matrix of interactions was made by mixing haploid cells of complementary mating types, harboring various BD-kinase and AD-kinase fusions, in a 96 wells format. Interaction phenotypes were assessed by the ability of the diploid forms to grow on selective media depleted for histidine. Positive interactants were identified by DNA sequencing.

### Sequence alignments and 3D-structure modeling

Sequence alignments were performed using MAFFT (Katoh and Toh, 2008). Structural models of PrkD (residues 17–253), YabT (residues 25 to 268), PtkA (residues 10 to 226), and HprK (residues 6 to 299) were obtained using the SWISS-MODEL (Bordoli et al., 2009). The residues 227 and 228 of PtkA were added to the structure manually to cover the autophosphorylation site Y228. Both PrkD and YabT were modeled based on PknB from *M. tuberculosis* (1mruB) (Young et al., 2003) as template. PtkA was modeled based on CapB from *Staphylococcus aureus* (2vedB) (Olivares-Illana et al., 2008), and HprK/P was modeled based on HprK/P from *Staphylococcus xylosus* (Márquez et al., 2002) (1ko7A). SpoIIAB structure has been experimentally resolved (1thnC) (Masuda et al., 2004).

## Results and discussion

### Cross-phosphorylation of bacillus subtilis serine/threonine and tyrosine kinases

**Figure 1:**
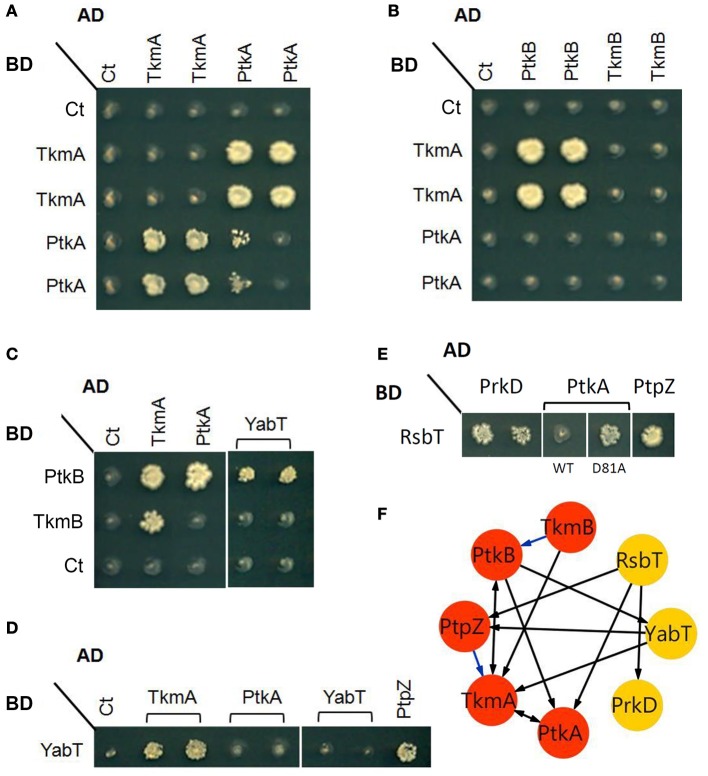
**Protein-protein interactions between some components of the tyrosine and serine/threonine signal transduction pathway**. Interaction phenotypes were monitored by the ability of the yeast cells co-expressing a given bait (Gal4 BD-fusion) and prey (Gal4 AD-fusion) pair of proteins to grow on selective media, as described in the experimental procedures. **(A)** Reciprocal interactions between PtkA and its cognate modulator TkmA (fused to either AD or BD). **(B)** Interactions among components of the two BY-kinase systems: PtkA/TmkA (fused to BD) and PtkB/TkmB (fused to AD). **(C–E)** Examples of interactions between different families of kinases: **(C)** In addition of TkmA (here as positive control), PtkB (fused to BD) interacts with PtkA and YabT (fused to AD). **(D)** YabT (fused to BD) interacts with TkmA and PtpZ (fused to AD). **(E)** RsbT (fused to BD) interacts PrkD, PtpZ and catalytically inactive PtkA (D81A) (fused with AD). **(F)** A graphical representation of the interactions revealed by yeast two-hybrid. Proteins (nodes) are linked by edges (arrows) directed from bait to prey. Blue edges indicate additional interactions found after yeast two-hybrid screenings of the *B*. *subtilis* genomic library (Shi et al., unpublished results).

In this study we set out to explore the possibility of cross-phosphorylation among eight *B*. *subtilis* protein kinases: PrkC, PrkD, YabT, PtkA, HprK/P, RsbT, RsbW, and SpoIIAB. The second BY-kinase PtkB (EpsB) (Gerwig et al., 2014) was left out from *in vitro* studies due to known solubility issues (Mijakovic et al., 2003). First we explored the capacity of the kinases to physically interact with each other, by performing two-hybrid screens using individual kinases as baits (Figure 1). An interaction detected between two proteins in both directions, i.e., irrespective of which one of them is used as a bait or prey, is termed reciprocal. An interaction is termed non-reciprocal when it has been detected with only one bait-prey configuration, but not the other. In our assay, we detected a reciprocal interaction between the BY-kinase PtkA and its cognate modulator TkmA, first described by Mijakovic et al. (2003) (Figure 1A). Next, we detected a reciprocal interaction between TkmA and the second BY-kinase PtkB (Figures 1B,C). There were also two non-reciprocal interactions: that of PtkA and PtkB and TkmA and TkmB (Figure 1C). The BY-kinases PtkA and PtkB were connected to the Hanks-type serine/threonine kinase YabT either via a direct interaction (PtkB, Figure 1C) or via an interaction with the PtkA modulator TkmA (Figure 1D). YabT also interacted with the phosphotyrosine-protein phosphatase PtpZ, known to dephosphorylate PtkA substrates (Mijakovic et al., 2005). The two-component-like kinase RsbT interacted directly with the BY-kinase PtkA and its cognate phosphatase PtpZ (Figure 1E). Interestingly, the RsbT did not interact with the wild-type PtkA, but interacted with the catalytically inactive mutant D81A. A direct interaction was also observed between RsbT and the Hanks-type kinase PrkD (Figure 1E). Hanks-type kinases YabT and PrkD also interacted directly with the sensory histidine kinase DegS which they are known to phosphorylate (Jers et al., 2011) (data not shown). Another link between tyrosine phosphorylation systems and the two-component systems was established through a binary interaction involving the phosphotyrosine-protein phosphatase YwlE (Musumeci et al., 2005) and the two-component response regulator YxdJ (Joseph et al., 2004) (data not shown). These findings suggest that physical interactions exist among three different classes of kinases: Hanks-type kinases, BY-kinases, and two-component systems-like kinases. The bi-functional kinase/phosphorylase HprK/P did not appear to interact with other kinases in the two-hybrid assay (data not shown). A weak growth phenotype, featuring a possible PtkA self-interaction was also observed in only one of the two tested AD-PtkA fusions (Figure 1A). This could be explained by differential levels of expression of the AD-PtkA fusions in the two different haploid yeast strains.

**Figure 2:**
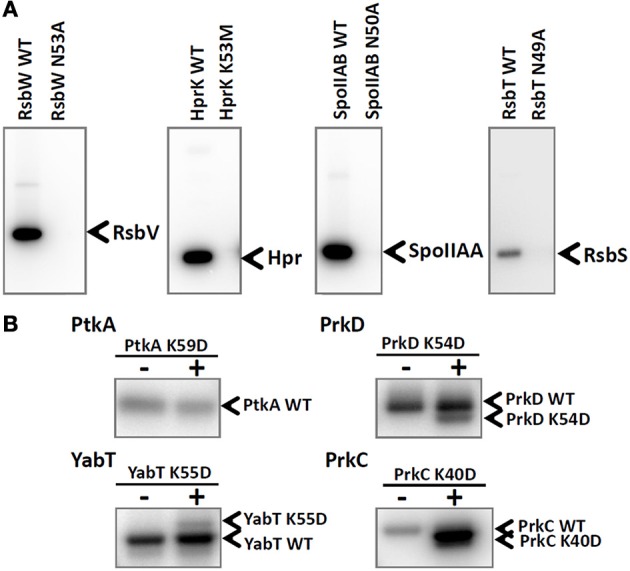
**Inactivation of catalytic residues in all tested *B. subtilis* kinases**. **(A)** Autoradiography images of the *in vitro* phosphorylation assays of RsbV, Hpr, SpoIIAA, and RsbS phosphorylated by their respective kinases: RsbW, HprK, SpoIIAB, and RsbT. Final protein concentrations in the assays were: 10 μM RsbV with 5 μM RsbW WT or RsbW N53A, 10 μM Hpr with 5 μM HprK/P WT or HprK K53M, 10 μM SpoIIAA with 1 μM SpoIIAB WT or SpoIIAB N50A, 3 μM RsbS with 3 μM RsbT WT or RsbT N49A. Bands corresponding to phosphorylated substrates are indicated by arrows. **(B)** Autoradiography images of intermolecular autophosphorylation assays (using incorporation of ^32^P) between wild type kinases PtkA, PrkC, PrkD, and YabT and their respective mutated versions PtkA K59D, PrkC K40D, PrkD K54D, and YabT K55D. Final protein concentrations in the *in vitro* phosphorylation assay were: 1 μM PtkA WT, 1 μM TkmA, 1 μM PtkA K59D, 1 μM PrkD WT, 1.5 μM YbdM K54D, 0.5 μM YabT WT, 0.5 μM YabT K55D, 0.5 μM PrkC WT, and 0.6 μM PrkC K40D. Wild type proteins were present on all lanes, and the presence or absence of mutant proteins is indicated with +/− above each lane. In order to be able to separate the WT kinases from the mutant versions, PtkA WT, PrkD WT, YabT K55D, and PrkC WT were fused with a 6xHis-tag and the respective mutant versions were Step-tagged fusions. Bands corresponding to phosphorylated kinases are indicated by arrows.

Next, we explored the capacity of purified protein kinases to phosphorylate each other. We first purified mutant versions of each kinase with inactivated catalytic residues. Since several of the studied kinases, HprK/P, RsbT, RsbW, and SpoIIAB, do not autophosphorylate, we tested their catalytically inactive mutant versions (HprK/P K53M, RsbT N49A, RsbW N53A, SpoIIA N50A) on their respective substrates: Hpr, RsbS, RsbV, and SpoIIAA (Figure 2A). All mutant kinases were unable to phosphorylate the substrates, indicating that the catalytic site inactivation has been completed successfully. Next, we examined the capacity for intermolecular autophosphorylation for the autophosphorylating kinases PtkA, PrkC, PrkD, and YabT (Figure 2B). In case of PrkC, PrkD and YabT, intermolecular autophosphorylation was indeed detected. The mutant versions PrkC K40D, PrkD K54D, and YabT K55D were all phosphorylated by their respective wild type counterparts, which is consistent with the know mode of activation of Hanks-type kinases through a binary interaction (Barthe et al., 2010). This signal of phosphorylation on PrkC K40D was particularly strong, suggesting that this kinase is very efficient in intermolecular autophosphorylation. Since PrkC is the only Hanks-type kinase in *B. subtilis* that possesses an extracellular ligand-binding domain, it seems plausible that its mode of activation follows the canonical trans-phosphorylation triggered by ligand-induced dimerization at the membrane. By contrast, YabT is known to be activated by binding DNA at the asymmetric septum during spore development (Bidnenko et al., 2013) and for the soluble PrkD, the activation mechanism is not known. For the BY-kinase PtkA we could not detect trans-autophosphorylation, which is in agreement with our previous findings (Mijakovic et al., 2003). For the remaining kinases, HprK/P, RsbT, RsbW and SpoIIAB, intermolecular autophosphorylation was not observed, as expected (data not shown).

**Figure 3:**
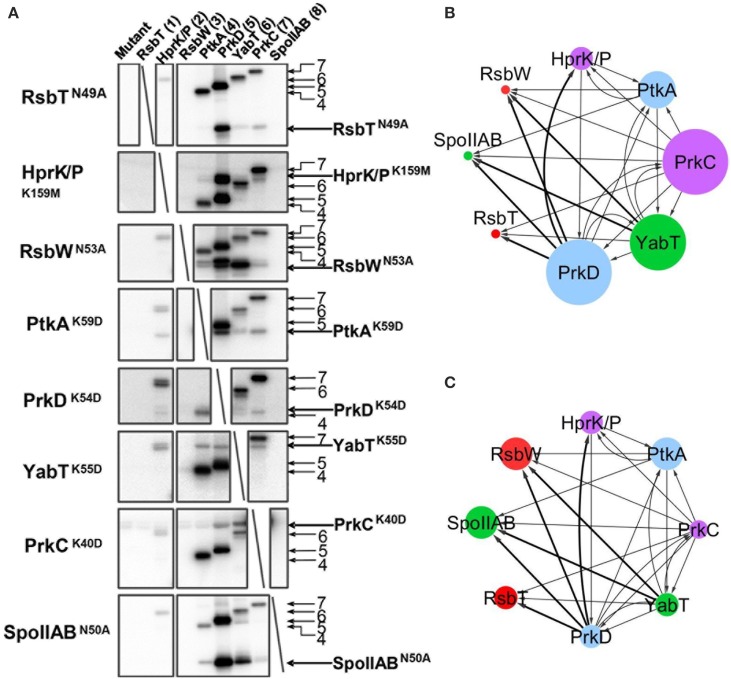
***In vitro* phosphorylation assay showing cross-phosphorylation among kinases**. Catalytically deficient protein kinase mutants carrying a substitution of the catalytic residue (indicated in each row) have been assayed pair-wise for phosphorylation by 8 different wild type kinases (indicated in columns and numbered) in the presence of ^32^P-γ-ATP. Signals from phosphorylated kinases have been revealed by autoradiography. First lane of each gel is the inactivated kinase substrate alone as a negative control. Bands corresponding to autophosphorylation of wild type kinases (numbered) and phosphorylation of mutated kinase substrates are indicated by arrows. **(B,C)** Graphical overview of the results shown in **(A)**. Panel **(B)** presents the outgoing degree of connectivity (number of phosphorylation reactions catalyzed by the kinase), and panel **(C)** shows the incoming degree of connectivity (number of phosphorylation reactions underwent by the kinase). The size of the nodes refers to the degree of connectivity of each kinase. Color of nodes refers to the physiological condition for up-regulation of kinase expression based on Nicolas et al. (2012): sporulation (green), germination (purple) oxidative stress (red), biofilm formation and swarming (blue). Connecting line width illustrates relative phosphorylation efficiency.

The catalytically deficient kinases were then subjected to cross-phosphorylation by the entire set of wild type kinases, in an eight by eight matrix experiment (Figure 3A). The reactions were assembled based on the assumption that kinases are present in roughly equimolar concentrations in the *B. subtilis* cell (Nicolas et al., 2012). Small variations in the final kinase concentrations in the assay are a result of optimization to simultaneously visualize signals of kinases migrating close to each other in SDS-PAGE. In this assay a number of cross phosphorylation events were detected. The results from the cross-phosphorylation assay are summarized in the kinase-kinase network (Figures 3B,C). Two main classes emerge with respect to propensity for cross-phosphorylation. The autophosphorylating kinases PrkD, PrkC, YabT, and PtkA (Figure 2A) exhibited a strong tendency to phosphorylate other kinases, i.e., they possess a high degree of outgoing connectivity (Figure 3B). PtkA and YabT reciprocally phosphorylated each other in agreement with their capacity to physically interact (Figure 1). The three kinases with the highest degree of incoming connectivity (i.e., propensity to be phosphorylated by other kinases) were SpoIIAB, RsbW, and PtkA (Figure 3C). The two-component-like kinases RsbW and SpoIIAB were found to be efficiently phosphorylated by the Hanks-type kinases PrkD and YabT. PrkD also efficiently phosphorylated HprK/P and RbsT. The latter observation correlates with our identification of the RsbT-PrkD interaction *in vivo* (Figure 1).

These findings suggest that cross-talk among different families of bacterial kinases is not only possible, but might in fact be quite common. As indicated in the previous section, our protein-protein interaction, and kinase-cross phosphorylation datasets exhibit a significant overlap. Connections between kinases which are supported by both approaches should be considered with a higher degree of confidence, but others should not be discarded. We have recently demonstrated that interactomics and phosphoproteomics datasets, while each detecting many kinase-substrate connections, fail to detect them all (Shi et al., unpublished results). Therefore, such complementary approaches should be combined whenever possible.

### Kinase cross-phosphorylation occurs at key regulatory residues

To explore the potential consequences of cross-phosphorylation on the recipient kinase function, we focused on several cases representing different types of cross-talk and identified the phosphorylated residues on recipient kinases by mass spectrometry. The examined “recipient” kinases: PtkA, PrkD, YabT, SpoIIAB, and HprK, comprised representatives of all four kinase types. After identifying the phosphorylation sites, we mapped them onto the resolved kinase structures or homology-based structural models, in order to evaluate the possible impact of cross-phosphorylation on the recipient kinase function.

**Figure 4:**
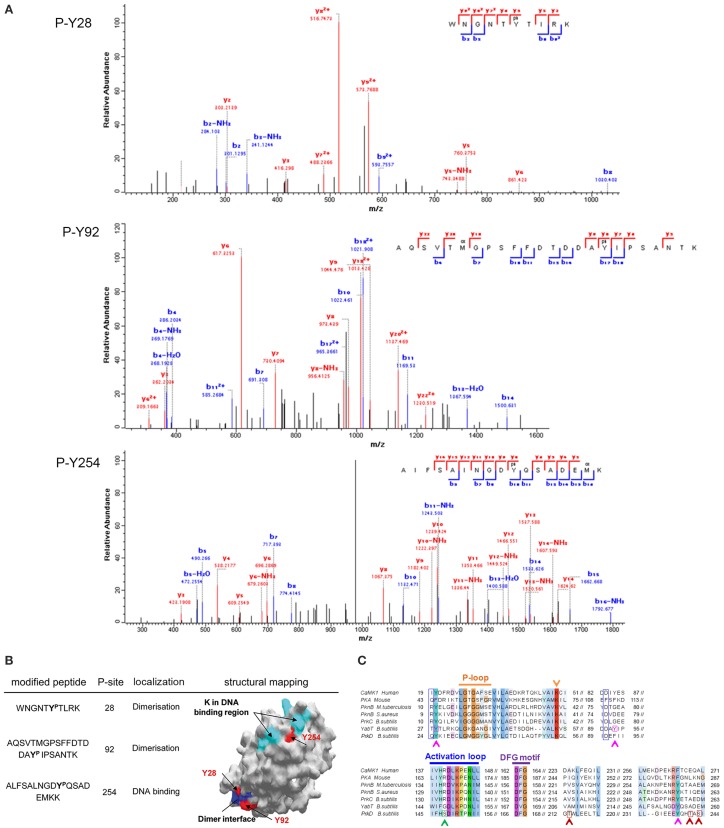
**Phosphorylation of YabT by PtkA**. **(A)** Identification of phosphorylated residues by mass spectrometry. Fragmentation spectra for the three modified peptides, bearing the phosphorylated tyrosine residues 28, 92, and 254, are shown. **(B)** Mapping of the phosphorylated residues on the structural model of YabT obtained by SWISS-MODEL (surface representation). The phosphorylated residues are shown in red, residues in blue are associated with the region involved in the formation of dimers in YabT and in PknB (Rakette et al., 2012) and the residues in cyan represent the DNA binding region of YabT (Bidnenko et al., 2013). **(C)** Alignment of *B. subtilis* YabT, PrkC, and PrkD sequences with Hanks-type kinase homologs: the kinase domains of calcium/calmodulin-dependent protein kinase (CaMK1, human), protein kinase A (PKA, mouse), PknB from *M. tuberculosis* and *Staphylococcus aureus*. The P-loop, catalytic site (K), catalytic loop and the DFG motif are indicated (Young et al., 2003). The residues R9 and D75 in PknB of *S. aureus* which are involved in dimer formation (Rakette et al., 2012) are indicated with blue boxes. For YabT, the residues Y28, Y92 and Y254, which are phosphorylated by PtkA, are indicated with pink boxes and arrows. For PrkD, the residues T213, T241, and S243, which is phosphorylated by PrkC, are indicated with red boxes and arrows, and the S148, which is phosphorylated by HprK/P, is indicated with a green box and arrow.

First we examined the phosphorylation of the Hanks-type kinase YabT by the BY-kinase PtkA. We detected three phosphorylated tyrosine residues on YabT (Figure 4A). Two of them (Y28 and Y92) are located in the cluster of conserved residues involved in dimer formation (Rakette et al., 2012) (Figure 4B). The third one (Y254) is embedded within a lysine/arginine-rich region essential for DNA binding by YabT, which is known to stimulate the kinase activity (Bidnenko et al., 2013) (Figure 4B). A negative charge introduced by phosphorylation at Y254 is likely to affect the YabT interaction with DNA, while phosphorylation at Y28 and Y92 might affect dimerization of the kinase. These findings clearly suggest that PtkA-dependent phosphorylation of YabT could modulate the activity of the recipient kinase. Sequence alignment of YabT with other Hanks-type serine/threonine kinases shows that the residues Y28 and Y254 in YabT are highly conserved (Figure 4C), suggesting that the regulatory mechanism involving their phosphorylation could be widely conserved.

**Figure 5:**
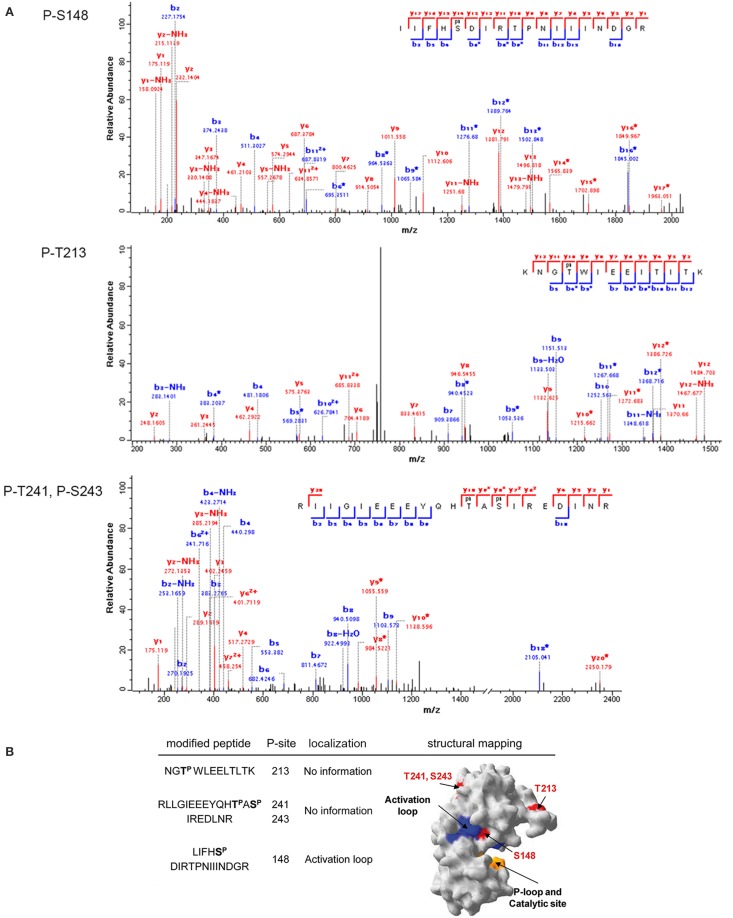
**Phosphorylation of PrkD by PrkC and HprK/P**. **(A)** Identification of phosphorylated residues by mass spectrometry. Fragmentation spectra are shown for the three modified peptides, bearing the residues threonine 213, threonine 241, and serine 243 phosphorylated by PrkC, and serine 148 phosphorylated by HprK/P. **(B)** Mapping of the phosphorylated residues on the structural model of PrkD obtained by SWISS-MODEL (surface representation). The phosphorylated residues are shown in red. Residues in the activation loop are in blue, and the catalytic site is in yellow.

PrkD is a cytosolic Hanks-type serine/threonine kinase with no transmembrane helix. For its transmembrane paralogs, PrkC and YabT, the activation mechanism by respective binding of muropeptides (Shah et al., 2008) and DNA (Bidnenko et al., 2013) has been clearly established. By contrast, the activation mechanism of PrkD is not known. We have detected that PrkD can be phosphorylated by PrkC and HprK/P. Phosphorylation of PrkD by PrkC occured on residues T213, T241, and S243 (Figure 5A), which are in the catalytic domain, but distant from the active site (Figure 5B). Any putative regulatory potential of these phosphorylation events can not be deduced directly. However, HprK/P phosphorylated PrkD on the residue S148 (Figure 5A) in the activation loop, with a very probable consequence of stimulating the PrkD kinase activity (Figure 5B). The residue serine 148 is not conserved in Hanks-type kinases (Figure 4C). The structure of PrkD, lacking the transmembrane helix, is also unusual for bacterial Hanks-type kinases. This configuration with another kinase phosphorylating the activation loop of PrkD (usually accomplished by intermolecular autophosphorylation leading to activation of canonical Hanks-type kinases) could represent an idiosyncratic mechanism to activate this cytosolic kinase.

**Figure 6:**
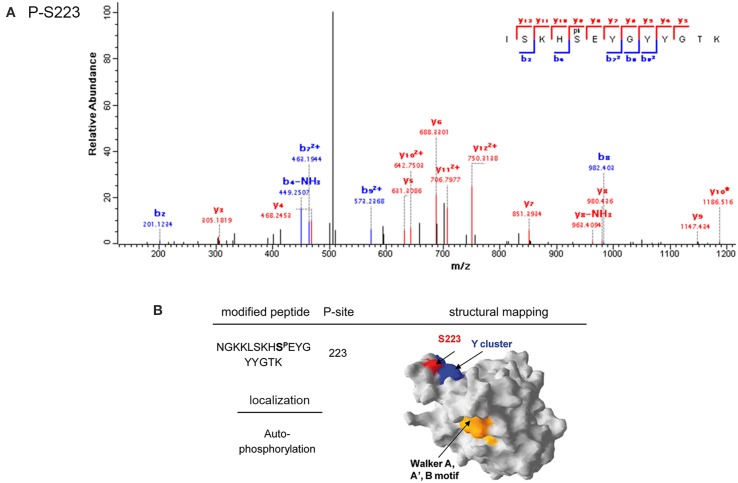
**Phosphorylation of PtkA by PrkC. (A)** Identification of the phosphorylated residue by mass spectrometry. Fragmentation spectrum for the peptide bearing the phosphorylated serine residue 223 is shown. **(B)** Mapping of the phosphorylated residues on the structural model of PtkA obtained by SWISS-MODEL (surface representation). The phosphorylated residue is shown in red. Residues in the C-terminal autophosphorylated tyrosine cluster are shown in blue, and the PtkA active site is in yellow.

The BY-kinase PtkA was phosphorylated by PrkC at S223 (Figure 6A). This residue is positioned in the immediate vicinity of the C-terminal tyrosine cluster, containing PtkA autophosphorylation sites (Y225, Y227, and Y228) (Figure 6B). BY-kinase autophosphorylation on these tyrosines is known to trigger the dissociation of the activator-bound octameric ring (Olivares-Illana et al., 2008). Interestingly, we have previously observed that PtkA autophoshorylation at Y228 is strongly enhanced *in vivo* in the Δ*prkC* strain (Ravikumar et al., 2014). This suggests that PrkC-dependent phosphorylation of PtkA at S223 could inhibit its autophosphorylation, and thus regulate its oligomerization state and interaction with the activator TkmA (Mijakovic et al., 2003).

**Figure 7:**
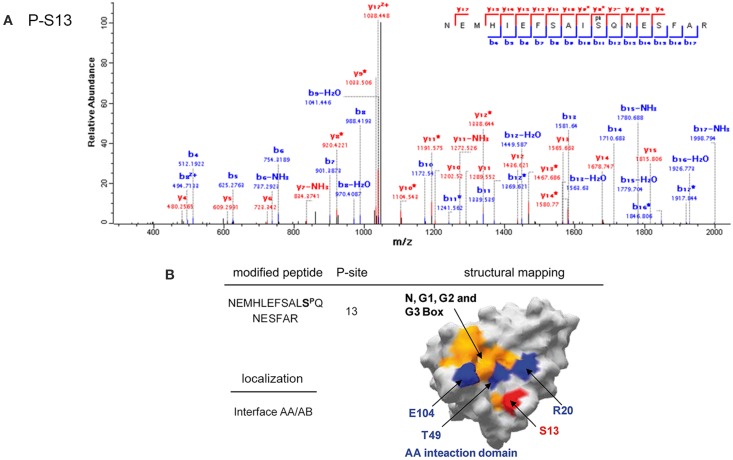
**Phosphorylation of SpoIIAB by PrkD. (A)** Identification of the phosphorylated residue by mass spectrometry. Fragmentation spectrum for the peptide bearing the phosphorylated residue serine 13 is shown. **(B)** Mapping of the phosphorylated residues on the resolved structure of SpoIIAB (Masuda et al., 2004) (surface representation). The phosphorylated residue is shown in red. The conserved boxes N, G1, G2, and G3, involved in ATP binding are highlighted in yellow (Campbell et al., 2002). Residues R20, T49 and E104, important for interaction with SpoIIAA (Campbell et al., 2002; Masuda et al., 2004) are highlighted in blue.

The two-component system-like serine/threonine kinase SpoIIAB was phosphorylated by PrkD at S13 (Figure 7A). This residue is located on the interface interacting with the anti-anti-sigma factor SpoIIAA (Figure 7B), close to the residues R20, T49, and E104, which are known to be essential for this interaction (Masuda et al., 2004). SpoIIAA is also an inhibitor of the SpoIIAB kinase activity, and must dissociate from the complex in order for the kinase to be active. Phosphorylation of SpoIIAB by PrkD at S13 might be an alternative route to destabilize the SpoIIAA/SpoIIAB complex, and its phosphorylation is therefore likely to have a regulatory role.

**Figure 8:**
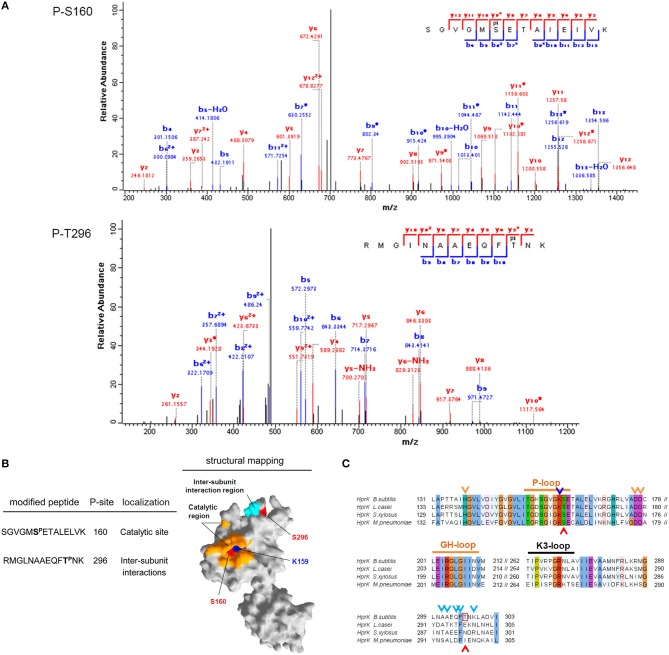
**Phosphorylation of HprK/P by PrkD. (A)** Identification of phosphorylated residues by mass spectrometry. Fragmentation spectra for the peptides with phosphorylated serine 160 and threonine 296 are shown. **(B)** Mapping of the phosphorylated residues on the resolved structure of HprK/P obtained by SWISS-MODEL (surface representation). The phosphorylated residues are shown in red, and the catalytic residue K159 is in blue. The catalytic site is highlighted in yellow, and the inter-subunit interaction region is shown in cyan. **(C)** Sequence alignment of HprK/Ps from *B. subtilis, Lactobacillus casei, Streptococcus xylosus*, and *M. pneumoniae*. Conserved motifs and residues in the catalytic region are highlighted. The catalytic residue K159 is indicated with blue arrow, and the amino acids involved in inter-subunit interactions (Márquez et al., 2002), are indicated with cyan arrows. The residues S160 and T296, which are phosphorylated by PrkD, are indicated with red boxes and arrows.

**Figure 9:**
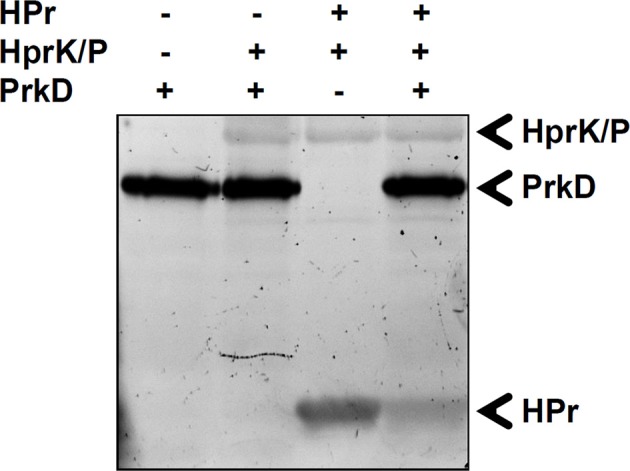
**Phosphorylation of HPr by phosphorylated HprK/P**. *In vitro* phosphorylation of HPr by HprK/P in the presence or absence of PrkD. Signals from phosphorylated protein have been revealed by Pro-Q^®^ Diamond phosphoprotein stain. The presence or absence of proteins is indicated as +/− above each lane. The phosphorylated bands corresponding to HprK/P, HPr, and PrkD are indicated by arrows.

Finally, the bifunctional HprK/P was phosphorylated by PrkD at residues S160 and S296 (Figure 8A). S296 is situated in the region necessary for subunit interactions in the HprK/P hexamer (Márquez et al., 2002) (Figure 8B), but it is not conserved (Figure 8C). By contrast, the phosphorylated S160 is situated adjacent to the catalytic residue K159 and is highly conserved (Figures 8B,C). We speculated that its phosphorylation is very likely to affect the catalytic activity of HprK/P, which depends on the positively charged catalytic lysine. In order to test this assumption, we phosphorylated HprK/P with PrkD *in vitro*, and then compared the capacity of phosphorylated vs. non-phosphorylated HprK/P to phosphorylate its substrate HPr (Figure 9). The presence of PrkD in the phosphorylation assay significantly diminished the capacity of HprK/P to phosphorylate HPr. The Pro-Q® Diamond-stained signal from phosphorylated HprK/P comprises the pre-existing artificial autophosphorylation of this kinase in its 6xHis tag (Josef Deutscher, personal communication), and therefore additional phosphorylation by PrkD is not readily discernable. While this finding supports the notion that HprK/P activity is regulated by PrkD-dependent phosphorylation, its physiological relevance remains to be determined *in vivo*.

### Conclusions and perspectives

Our global interactomics screen of the regulatory network based on serine/threonine/tyrosine phosphorylation in *B. subtilis* (Shi et al., unpublished results) revealed a high degree of connectivity among different classes of kinases, kinase activators, substrates, and phosphatases. Here we have explored whether the connectivity at the protein-protein interaction level may point to a functional interaction, i.e., cross-phosphorylation among the four classes of *B. subtilis* serine/threonine and tyrosine protein kinases. Our data suggest an even higher degree of connectivity than the interactomics studies, with all tested kinases engaging in cross phosphorylation, either as donors or recipients. Moreover, the identity of residues phosphorylated on recipient kinases in most cases supports the notion that phosphorylation of one kinase by another has a functional/regulatory consequence. The phosphorylated residues are situated in, or immediately adjacent to, protein-protein or protein-DNA interaction surfaces, activating loops or the kinase active sites. It is therefore likely that bacteria also possess kinase cascades similar to those described in eukarya (Marshall, 1994). Given the existing evidence on involvement of these kinases in complex phenomena such as bacterial cell cycle control and control of spore and biofilm development, this finding is not entirely unexpected. The kinase phosphorylation sites that we report here were not detected in the previous phosphoproteomics studies performed on *B. subtilis* (Macek et al., 2007; Ravikumar et al., 2014), which is not surprising due to the non-exhaustive nature of published bacterial phosphoproteomes. Nevertheless, the final verdict on the importance of kinase cross-phosphorylation and the putative kinase cascades in bacteria will have to come from *in vivo* studies.

### Conflict of interest statement

The authors declare that the research was conducted in the absence of any commercial or financial relationships that could be construed as a potential conflict of interest.
